# 农产品中脱氧雪腐镰刀菌烯醇的表面增强拉曼检测

**DOI:** 10.3724/SP.J.1123.2022.06021

**Published:** 2022-11-08

**Authors:** Mingming CHEN, Bihang SU, Jianli HUANG, Fengfu FU, Yongqiang DONG

**Affiliations:** 1.福州大学化学学院, 食品安全与生物分析教育部重点实验室, 福建省食品安全分析与检测技术重点实验室, 福建 福州 350108; 1. MOE Key Laboratory for Analytical Science of Food Safety and Biology, Fujian Provincial Key Laboratory of Analysis and Detection Technology for Food Safety, College of Chemistry, Fuzhou University, Fuzhou 350108, China; 2.福建省粮油质量监测所, 福建 福州 350108; 2. Fujian Grain and Oil Quality Monitoring Institute, Fuzhou 350108, China

**Keywords:** 表面增强拉曼散射, 单层碳基点, 银纳米颗粒团聚体, 水凝胶, 脱氧雪腐镰刀菌烯醇, surface-enhanced Raman scattering (SERS), single-layer carbon-based dots (CDs), aggregated silver nanoparticles (a-AgNPs), hydrogel, deoxynivalenol (DON)

## Abstract

利用便携式拉曼光谱仪建立了一个快速筛查与检测谷物中真菌毒素脱氧雪腐镰刀菌烯醇(DON)的表面增强拉曼散射(SERS)方法。首先利用实验室前期开发的方法制备了具有高活性的水凝胶SERS芯片。该SERS芯片是将预先制备的高SERS活性的单层碳基点(CDs)包裹的银纳米颗粒团聚体(a-AgNPs/CDs)与聚乙烯醇(PVA)水溶液混合均匀后,再利用循环冷冻-解冻的物理交联法制备而成的。实验优化了影响水凝胶SERS芯片对DON的SERS响应的实验条件,包括溶剂、浸泡温度和浸泡时间。在最佳的SERS检测条件下(溶剂为水-乙醇(1:1, v/v),浸泡温度为40 ℃,浸泡时间为5 min), DON的线性响应范围为1~10000 μg/kg(相关系数(*R*^2^)=0.9967),检出限(LOD)为0.14 μg/kg,表明该SERS基底具有较高的灵敏度。得益于水凝胶特殊的孔径结构,实际样品基质中常见的糖、蛋白质、油脂、色素等干扰物质都被阻隔在水凝胶外。因此,在复杂样品检测中仅需要简单的提取,而不需要复杂的分离处理。将该方法用于小麦粉中DON的检测,所得回收率为97.3%~103%,相对标准偏差为4.2%~5.0%。实验结果表明所建立的检测DON的SERS方法具有响应范围宽、灵敏度高、重复性好、响应迅速、操作简单、抗干扰能力强等优点,这说明本实验室所构建的水凝胶SERS芯片在粮食中生物毒素的快速筛查与检测方面具有良好的应用潜力。

## Abstract (en)

Fungal toxins are secondary metabolites of fungi. Food is highly susceptible to contamination by various fungal species that produce fungal toxins during production and storage. Fungal toxins can cause either acute or chronic poisoning from long-term, low-dose ingestion. Therefore, fungal toxins have become a topic of international interest as a food safety issue. Deoxynivalenol (DON) is a single-terminal sporam toxin produced predominantly by Fusarium graminae and Fusarium pinkosa. DON is globally one of the most common fungal toxins contaminating grain, food, and feed. Various methods have been applied for screening and detecting DON; however, these methods utilize expensive instruments and entail complex operations, poor repeatability, and low sensitivity. Therefore, the development of a simpler, more rapid, and sensitive sensing technology for DON detection is important for applications within the agriculture and food industry. Recently, surface-enhanced Raman scattering (SERS) has become a rapidly developing spectral analysis technology with unique advantages, including high sensitivity, high throughput, and rapid response rates. Therefore, attempts have been made to apply the SERS technique to detecting DON. However, due to the limitations concerning SERS substrates, the currently established SERS method exhibits serious problems, including low sensitivity and weak anti-interference ability, and cannot meet the requirements of sample detection. Recently, our group has prepared aggregated silver nanoparticles (a-AgNPs/CDs) with high SERS activity by using single-layer carbon-based dots (CDs) as a capping agent. Moreover, the obtained materials (a-AgNPs/CDs) were combined with hydrogel technology to prepare novel hydrogel SERS chips. The obtained SERS chips exhibited several advantages over traditional SERS substrates, such as high sensitivity, long-term stability, improved uniformity, and strong anti-interference capabilities. Herein, a novel SERS method for rapid screening and detection of DON in grains was established using a portable Raman spectrometer based on the developed hydrogel SERS chips. The main experimental conditions were optimized before the SERS detection of DON; this included the optimization of the hydrogel SERS chip soaking temperature and time in the DON solution. It was found that the optimal soaking temperature and time were 40 ℃ and 5 min, respectively. Under the optimal SERS detection conditions, the linear response range of DON was 1-10000 μg/kg (correlation coefficient (*R*^2^)=0.9967), and the limit of detection (LOD) was 0.14 μg/kg. Due to the unique pore size structure of the hydrogel, common sugar, protein, oil, pigment, and other interfering substances in the sample matrix were blocked outside the hydrogel. Therefore, only simple extraction was required while detecting complex samples. This method was applied to detecting DON in wheat flour, yielding recoveries of 97.3%-103% with relative standard deviations of 4.2%-5.0%. The established SERS method for DON detection exhibits a broader response range, high sensitivity, good repeatability, rapid response, simple operation, and strong anti-interference capability. This shows that the laboratory-constructed hydrogel SERS chip has excellent potential for rapid screening and detection of biotoxins in food.

真菌毒素是真菌的次级代谢产物。粮食在生产和储藏过程中都极易受到不同种类真菌的污染,从而产生真菌毒素。真菌毒素既可引起急性中毒,也可由于长期低剂量摄入引起慢性中毒。有些真菌毒素甚至还具有致突变性、致癌性、致畸性以及对特定系统与器官的毒性,如神经系统毒性、生殖系统毒性、肾毒性、出血性毒性、肝毒性和免疫抑制毒性^[1,2]^。而且大部分真菌毒素具有很强的热稳定性,即使在食物煮熟后,其中所含有的真菌毒素仍稳定存在。因此,真菌毒素已经成为国际关注的食品安全热点问题之一。

脱氧雪腐镰刀菌烯醇(deoxynivalenol, DON)是一种单端孢霉烯族毒素,主要由禾谷镰刀菌和粉红镰刀菌产生。在全世界范围内,DON是最常见的一种污染粮食、饲料和食品的霉菌毒素之一。在中国,每年超过700万公顷的小麦受镰刀菌头枯病影响,从而导致大量小麦受到DON毒素的污染。食用被DON污染的小麦籽粒及其制品的动物和人类会出现急性中毒症状,如厌食、呕吐、腹泻、发烧和造血系统受损,甚至死亡^[3]^。因此世界各国对粮食和饲料中的DON都有非常严格的限量标准。我国规定大麦、小麦、燕麦、青稞、玉米原粮谷物中DON限量为2000 μg/kg。目前关于粮食中DON的检测方法以仪器分析法为主,包括液相色谱法(LC)、气相色谱-质谱法(GC-MS)和液相色谱-质谱法(LC-MS)等^[4,5]^。除仪器分析方法外,酶联免疫分析法、生物传感器等也可用于真菌毒素的分析^[6⇓-8]^。这些方法往往都具有很好的灵敏度,可完美地实现对真菌毒素的定量检测。然而,这些方法又都存在着一定的局限性,比如仪器分析方法存在设备昂贵、对操作者的技术要求高,且容易受到杂质干扰等缺点;免疫法容易出现假阴性和假阳性问题,而且稳定性也通常较差;生物传感技术经常出现交叉反应、重现性低等问题。另外,这些方法普遍存在着耗时、过程复杂等操作层面上的问题。探索一种更为简单、迅速、灵敏的用于DON检测的传感技术对于农业与食品业发展而言具有非常重要的意义。

表面增强拉曼散射(surface-enhanced Raman scattering, SERS)是近年来迅速发展起来的一种光谱分析技术^[9]^,具有高灵敏度、高通量、响应快速等独特优势。因此在表面科学^[10,11]^、材料科学^[12]^、生物医学^[13,14]^、药物分析^[15⇓-17]^、食品安全^[18⇓-20]^、环境检测^[21]^等领域显示出巨大的应用潜力。目前已经有人尝试利用SERS技术对食品中的DON进行快速检测,但从其结果来看,仍然存在灵敏度差、重现性不好、抗干扰能力弱等问题,因此不能广泛应用于实际样品的检测^[22⇓-24]^。这主要是由其SERS基底自身问题所决定的。众所周知,SERS测试需要将被测分子吸附在一些特殊材料(即所谓的基底材料)的表面。在电磁增强(EM)和/或化学增强(CM)的作用下,被测分子的拉曼信号大大增强。因此,SERS测试的结果在很大程度上取决于基底的性质。然而,目前报道的SERS基底中很少能同时兼顾高灵敏度、良好的均匀性和良好的稳定性。近年来,有人提出将SERS活性材料与水凝胶结合,有望获得具有良好均匀性和稳定性的SERS基底^[25]^。受此启发,我们制备了具有高SERS活性的聚集态碳点包裹银纳米颗粒(a-AgNPs/CDs)和聚集态金纳米颗粒,并将其嵌入水凝胶中,制备水凝胶SERS芯片^[26]^。所获得的SERS芯片具有灵敏度高、稳定期长、均匀性好、抗干扰性强等优点。这一结果将大大促进SERS技术在定量分析中的应用。本工作利用本实验室开发的a-AgNPs/CDs-水凝胶SERS芯片,构建粮食中DON快速检测的SERS传感方法,并应用于实际样品。该方法具有灵敏度高、线性范围宽、重复性和回收率好、抗干扰能力强等优点。

## 1 实验部分

### 1.1 仪器、试剂与材料

高分辨透射电子显微镜(HRTEM, 2100, JEOL,日本);场发射扫描电镜(FE-SEM, 6700F, JSE,日本);紫外-可见分光光度计(UV-Vis, Lambda 750, PerkinElmer,美国);便携式拉曼光谱仪(i-Raman, BWS465-532H, BW&TEK,美国)。

硝酸银(AgNO_3_,纯度99.0%)、聚乙烯醇(PVA, [C_2_H_4_O]*_n_*,聚合度1799,纯度98.0%~99.0%)购于上海阿拉丁生化科技有限公司;葡萄糖(C_6_H_12_O_6_,分析纯)和结晶紫(crystal violet, CV,分析纯)购于上海麦克林公司;氢氧化钠(NaOH,纯度96.0%)购于宜昌西隆化工有限公司;去离子水由Milli-Q纯水系统获得,并用于制备所有储备液和工作溶液。

### 1.2 实验条件

#### 1.2.1 a-AgNPs/CDs的合成

具有高SERS活性的a-AgNPs/CDs是以单层碳基点(carbon-based dots, CDs)为包裹剂^[27]^,采用一锅自组装方法合成的^[28]^。将1.2 mL CD溶液(5 mg/mL)和0.6 mL AgNO_3_溶液(1 mol/L)分散到50 mL去离子水中,随后用NaOH(0.1 mol/L)溶液调节其pH到9。在120 ℃油浴锅中回流15 min后加入0.6 mL的葡萄糖溶液(90 mg/mL),反应20 min之后停止加热,在搅拌下将溶液自然冷却到室温。随后,将冷却后的混合物用去离子水离心洗涤3次(12000 r/min,每次10 min)。最后,将得到的沉淀物(a-AgNPs/CDs)重新分散在10 mL去离子水中,并储存在4 ℃以备进一步使用。

#### 1.2.2 水凝胶SERS芯片的合成

水凝胶SERS芯片的制备方法按照本实验室最近发表的一项研究工作^[26]^进行。将1.4 g PVA和9.6 mL去离子水混合并在90 ℃下搅拌1.5 h,直到PVA完全溶解。将PVA溶液冷却到室温后加入1 mL a-AgNPs/CDs溶液(3 mg/mL),接着搅拌30 min。最后,将混合物转移到一个厚度为1 mm的玻璃模具中,通过在-24 ℃和室温下循环冷冻-解冻5次得到水凝胶SERS芯片。

#### 1.2.3 标准溶液的制备和SERS测量

用水-乙醇(1:1, v/v)作为溶剂配制一系列的DON标准溶液(10000、1000、100、50、10、5和1 μg/kg)。SERS检测前将0.5 mm×0.5 mm的水凝胶SERS芯片浸泡于50 μL的DON标准溶液中一段时间,然后取出,并在每个芯片上随机选取10个点进行测量。

#### 1.2.4 样品前处理

称取2 g加标的小麦粉样品于离心管中,加入10 mL水-乙醇(1:1, v/v),涡旋混匀。于室温下超声提取10 min后以12000 r/min离心10 min,收集上清液并转移到另一个干净的离心管中,向残渣中再加入10 mL乙醇-水(1:1, v/v)重复提取一次,合并上清液,得到小麦标准提取液。

#### 1.2.5 拉曼条件

便携式拉曼光谱仪拉曼激光为532 nm(BWS465-532H);激光功率为5 mW;单次积分时间为30 s;浸泡后的水凝胶SERS芯片表面无需冲洗,直接用于室温下的拉曼检测。

## 2 结果与讨论

### 2.1 材料的表征

通过TEM和FE-SEM表征a-AgNPs/CDs的形貌(见图1a和b)。所制备的AgNPs/CDs纳米颗粒粒径分布均匀,直径约为20 nm。a-AgNPs/CDs主要是由几十个AgNPs/CDs在二维方向上组成的聚集体。每个聚集体中相邻粒子之间的间隙在2 nm以下,因此,a-AgNPs/CDs具有丰富的电磁场“热点”。通过UV-Vis表征(见图1c)发现a-AgNPs/CDs呈现出两个特征的局域表面等离子体共振(localized surface plasmon resonance, LSPR)吸收峰,一个在395 nm,另一个在605 nm。

**图1:**
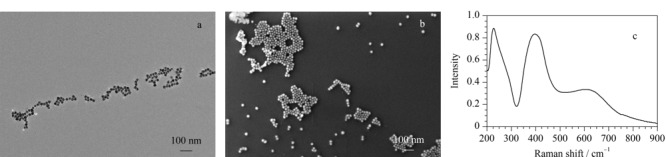
a-AgNPs/CDs的(a)透射电镜图、(b)扫描电镜图以及(c)紫外-可见吸收光谱

### 2.2 水凝胶SERS芯片基底的全面评估

图2a展示了实验所得的水凝胶SERS芯片。实验中以CV作为探针分子对所得水凝胶SERS芯片的性能进行评估。如图2b所示,CV的SERS强度随其浓度的增加而逐渐增加,并且在10^-12^~10^-16^mol/L的范围内,浓度的对数与其特征信号强度(1623 cm^-1^)呈现良好的线性关系,其线性方程为*Y*=11077 log *C*_CV_+181041,相关系数(*R*^2^)为0.9986。另外,所得水凝胶SERS芯片还具有非常良好的均一性。在芯片上40×40 μm^2^的范围内随机采集100个位点上的SERS光谱,可以清楚地发现所得的100条SERS光谱没有明显的差异(图2c)。光谱中1623 cm^-1^处峰强度构建的成像很均匀,没有明显的波动(图2d),据此计算得到的RSD为1.5%,远远低于大多数已报道的SERS基底。这些结果表明,所得水凝胶SERS芯片具有出色的灵敏度、均一性,因此可用于后续SERS检测。

**图2:**
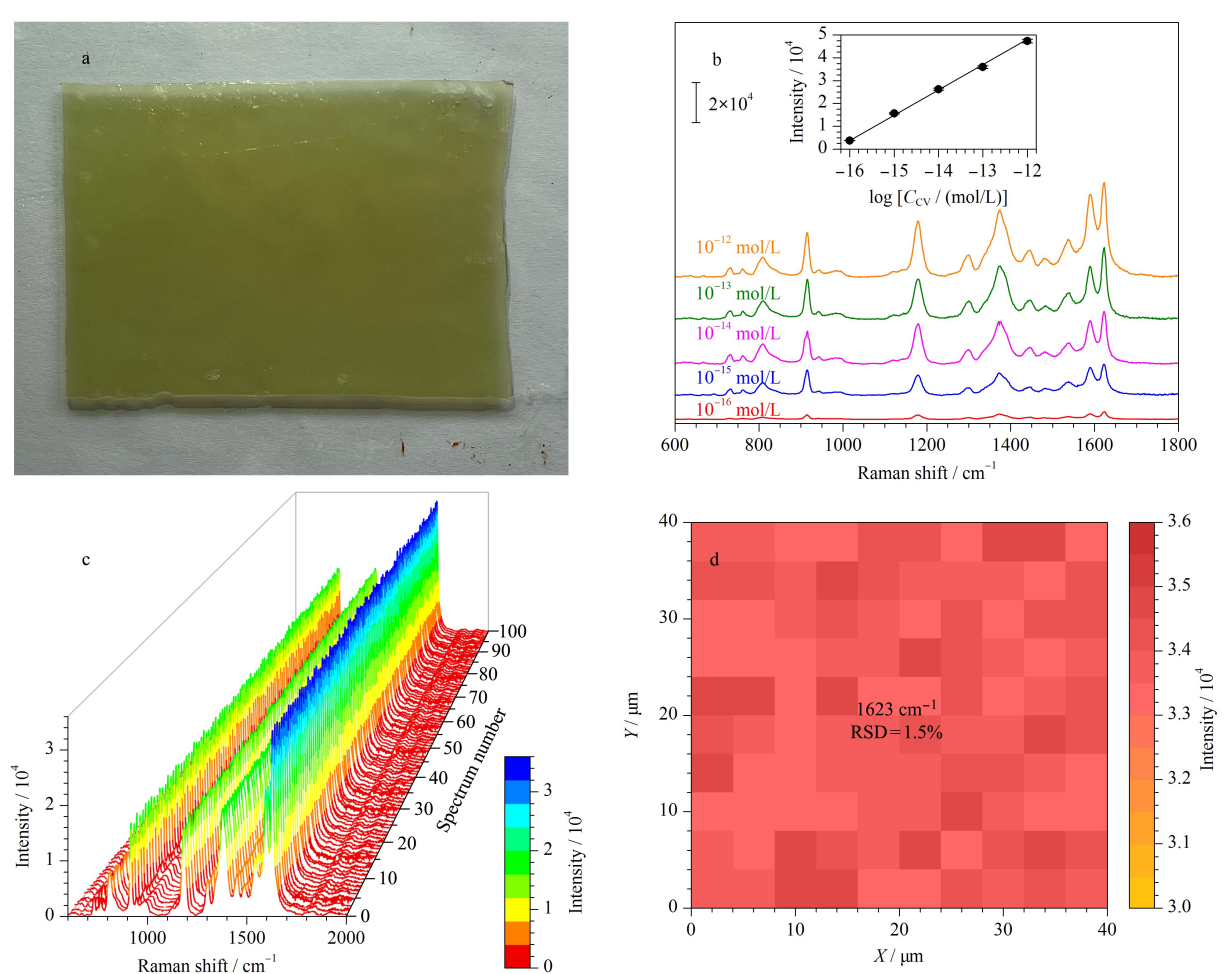
(a)水凝胶SERS芯片在可见光下的照片, (b)水凝胶SERS芯片对不同浓度CV的SERS响应, (c)从水凝胶SERS芯片上采集 100个CV(10^-13^mol/L)的SERS光谱, (d)100条收集到的SERS光谱中CV在1623 cm^-1^处的SERS信号的拉曼映射图

### 2.3 溶剂对水凝胶SERS芯片检测DON的影响

DON是一种无色针状固体,可溶于水和极性溶剂,如含水甲醇、含水乙醇或含水乙酸乙酯。本文比较了3种不同极性的溶剂水-甲醇(1:1, v/v)、水-乙醇(1:1, v/v)、水-乙酸乙酯(1:1, v/v)对水凝胶SERS芯片检测DON的影响。如图3a所示,结果显示当溶剂为水-乙醇(1:1, v/v)时,DON的SERS信号最强。随后比较了不同体积比(3:1、1:1和1:3)的水-乙醇对水凝胶SERS芯片检测DON的影响。实验条件优化如图3b所示,当溶剂水-乙醇体积比为1:1时,水凝胶SERS芯片检测DON得到了最大的增强。所以,以水-乙醇(1:1, v/v)为最终实验条件用于后续的实验研究。

**图3:**
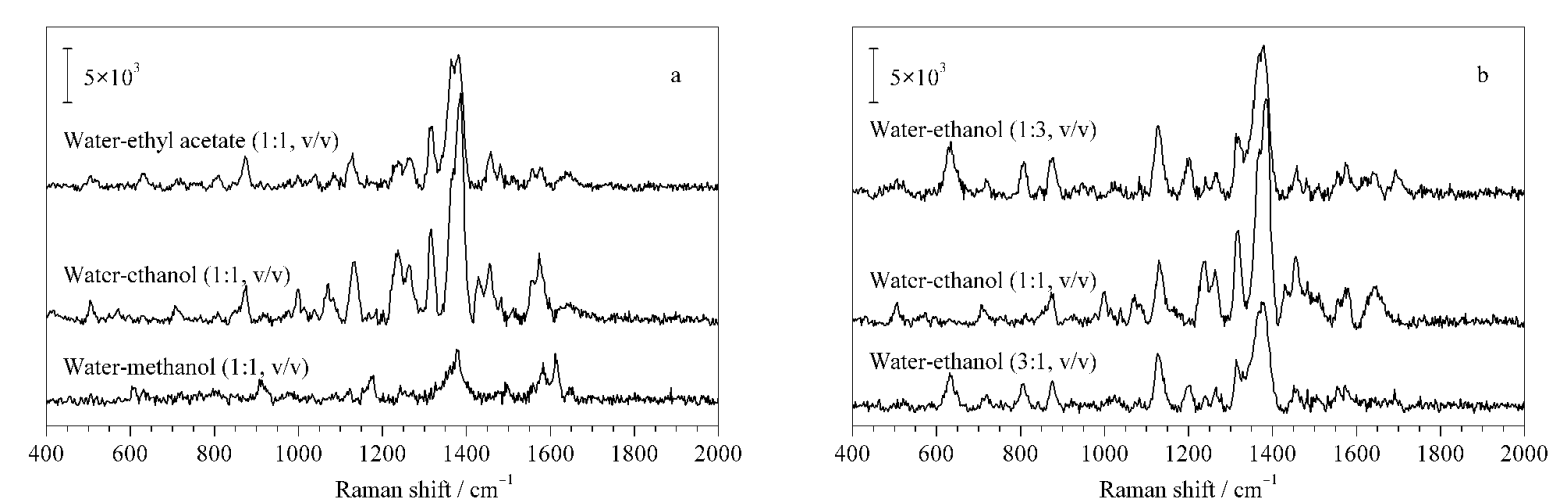
(a)不同溶剂和(b)不同水-乙醇体积比对水凝胶SERS芯片所得DON(10000 μg/kg)拉曼光谱的影响

### 2.4 浸泡温度及浸泡时间对水凝胶SERS芯片检测DON的影响

众所周知,通过SERS技术检测实际样品中的小分子时,样品基质中各种大分子(如多肽、蛋白质、多糖、寡核苷酸等)和油脂都具有很强的干扰作用。水凝胶具有有限大小的多孔网络结构,允许流体动力学直径小于孔隙大小的分子进入^[25]^。因此,水凝胶SERS芯片可具有出色的抗干扰能力。然而,待测小分子进入水凝胶的核心需要一段时间,这取决于分子扩散的速度。因此,在利用水凝胶SERS芯片对DON进行检测前需对浸泡温度和浸泡时间做相应的考察优化。

如图4a所示,不同浸泡温度下,DON的SERS光谱几乎没有区别,但是信号强度上却有明显的变化。如图4b所示,在较低温度下(40 ℃以下),小分子的扩散速度随着温度升高而变快,在40 ℃时DON的SERS信号达到最大值。在较高温度时(40 ℃以上),虽然小分子的扩散速度也会进一步加快,但是高温一定程度上破坏了水凝胶的三维结构,DON的SERS信号反而随着温度的升高而有所下降。因此,选择最佳浸泡温度为40 ℃。如图5a所示,在其他实验条件保持不变的情况下,延长水凝胶SERS芯片在DON溶液中的浸泡时间也可以明显地提高其SERS信号。从图5b可以看出,DON在1380 cm^-1^处的特征信号强度在3 min之内随浸泡时间增加而迅速增加,随后增加速度逐渐变缓。当浸泡时间超过5 min时,其信号强度逐渐趋于稳定。因此,选择最佳浸泡时间为5 min。

**图4:**
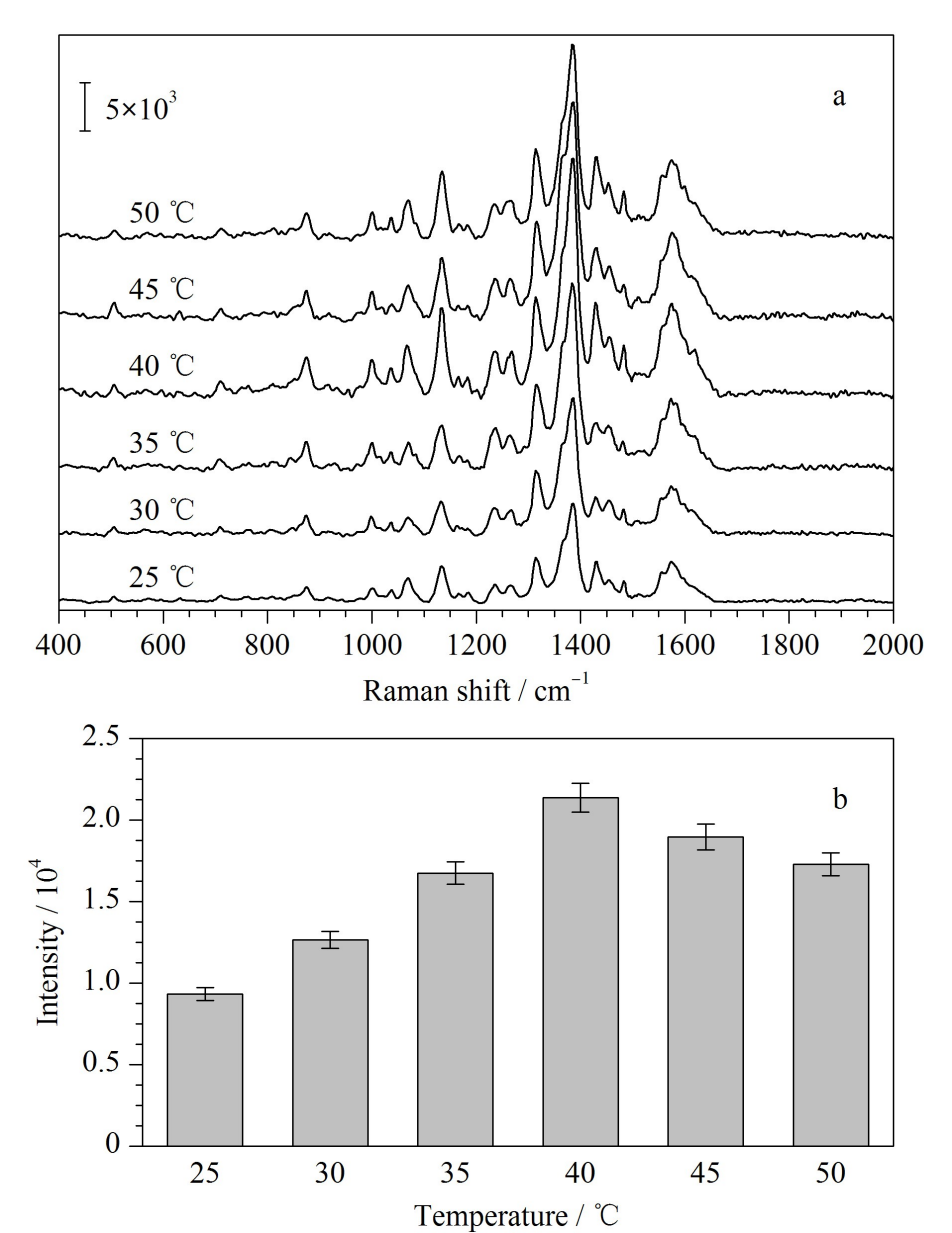
(a)水凝胶SERS芯片在DON溶液(10000 μg/kg)中浸泡温度对所得拉曼光谱的影响及(b)浸泡温度对DON在1380 cm^-1^处峰强度的影响(*n*=5)

**图5:**
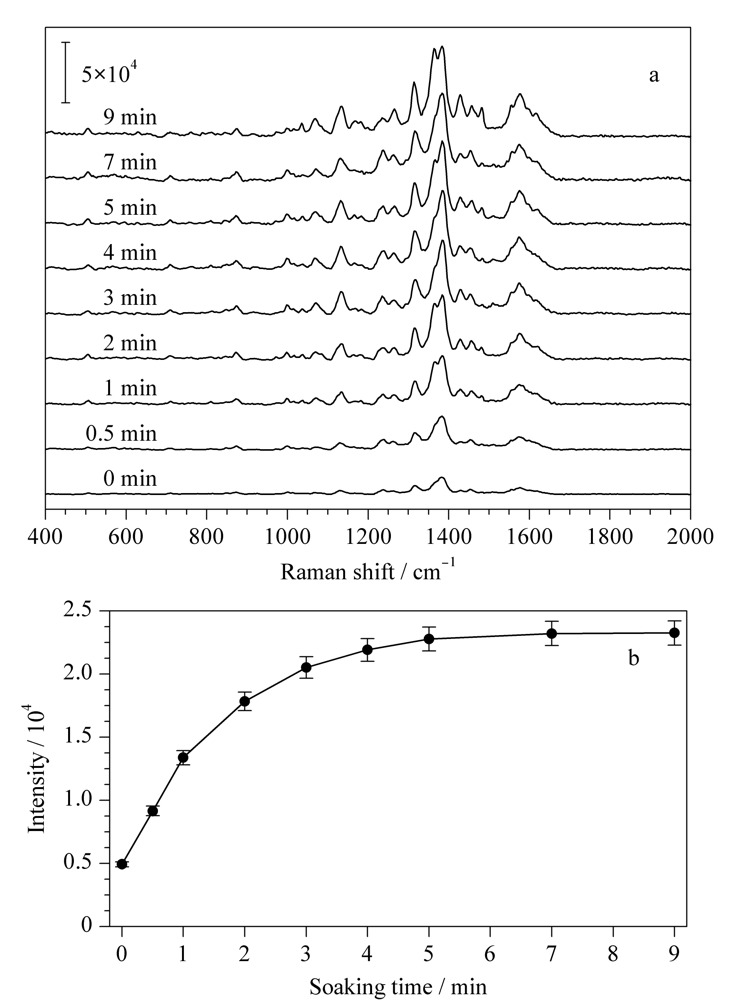
(a)不同浸泡时间下DON(10000 μg/kg)在水凝胶SERS芯片上的SERS光谱图和(b)浸泡时间对DON在1380 cm^-1^处峰强度的影响(*n*=5)

### 2.5 线性范围、检出限以及定量限

根据上述实验结果,利用所得水凝胶SERS芯片在最优条件下对不同含量的DON进行SERS检测。图6a展示了不同含量DON在所用水凝胶SERS基底上的SERS光谱。从图中可以看出即使DON的含量低至1 μg/kg,其SERS光谱中的主要特征峰仍可清楚分辨。当DON的含量从1 μg/kg逐渐增加到10000 μg/kg,其SERS光谱的形状没有发生明显的变化,主要特征峰的拉曼位移也没有发生变化。然而,所有特征峰的强度都随着DON含量的提高而明显增强。如图6b所示,DON在1380 cm^-1^处的SERS强度(*Y*)与DON的含量(*C*, μg/kg)的对数成正比,其线性方程为*Y*=5789 log *C*+1043, *R*^2^为0.9967。

**图6:**
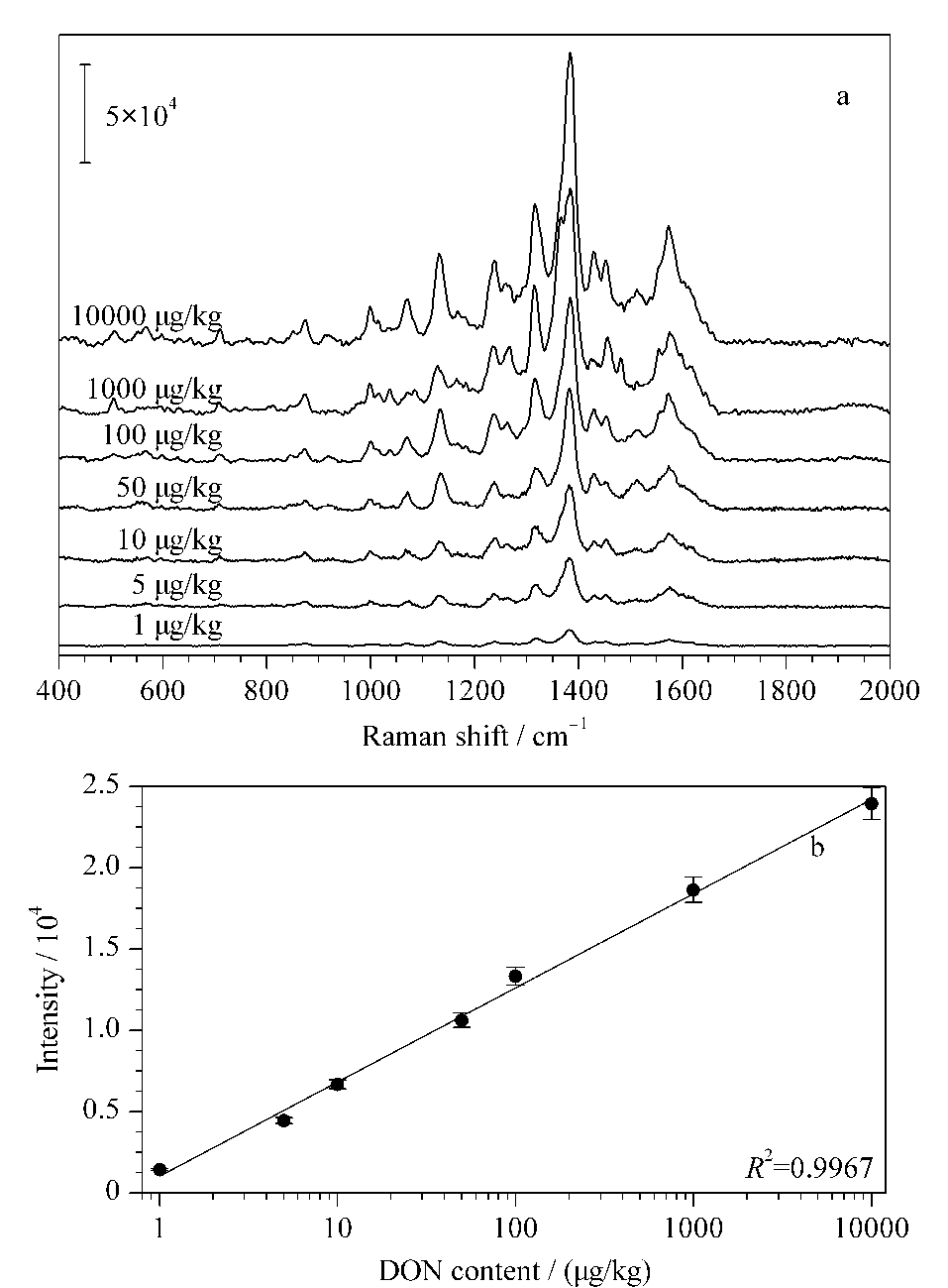
(a)不同含量DON在水凝胶SERS芯片上的SERS光 谱图和(b)DON在1380 cm^-1^处的特征峰强度与其含量对数的关系曲线

根据SERS光谱的特征峰计算检出限(LOD)和定量限(LOQ),见公式(1)和(2)。


(1) $LOD=\frac{3S_{k}}{K}$

(2) $LOQ=\frac{10S_{k}}{K}$

其中*S*_k_为空白样本光谱强度的标准差,*K*为拟合关系曲线的斜率。得到的结果分别为LOD 0.14 μg/kg和LOQ 0.47 μg/kg。将该方法与已有报道的DON的SERS检测方法进行比较(见表1),该方法具有更宽的线性范围和更低的检出限,说明该方法具有一定的优越性。

**表1:** 本研究方法与其他已报道SERS方法的比较

Material	Linear range/ (μg/kg)		LOD/ (μg/kg)	Application	Ref.
Cit-AgNPs^a^	29.6-	2963000	29.6	no detected	[23]
Cit-AuNPs^b^	500-	50200	110	wheat kernel	[20]
AAO-PDMS-Au^c^	50-	10000	24.8	maize	[18]
Hydrogel SERS	1-	10000	0.14	wheatmeal	this
chip					work

a. citrate-stabilized silver nanoparticles; b. citrate-stabilized glod nanoparticles; c. Au nanoparticles (Au NPs) on the surface of polydimethylsiloxane coated anodic aluminum oxide (PDMS@AAO) complex substrate.

### 2.6 实际样品检测

为了进一步考察所构建的SERS检测方法的实用性,我们将其应用于小麦粉中DON的检测。实验结果表明,小麦粉提取液中未检测到DON的SERS信号,说明小麦粉样品中DON的含量低于该方法的检出限。因此,实验采用空白样品加标的方式对小麦粉中DON的回收率进行考察。分别于小麦粉中加入低、中、高3个水平的DON,再按照1.2.4节中所述的方法将其提取出来。用所得的水凝胶SERS芯片对提取液平行测定3次,并根据建立的标准工作曲线计算平均回收率。由于水凝胶SERS芯片具有特殊的孔径结构,在复杂样品基质检测中可以隔绝大分子(糖、色素、油脂、蛋白质等),加之SERS是一种可以检测和鉴别分子、可反映结构信息的光谱技术,因此水凝胶SERS芯片可以在高灵敏度下仍然保持高选择性。结果见表2, DON在小麦粉中的平均回收率为97.3%~103%,符合方法验证规范要求的85%~115%。RSD为4.2%~5.0%,同样符合方法验证规范≤10%的要求。

**表2:** 小麦粉中DON的加标回收率(*n*=3)

Added/(μg/kg)	Found/(μg/kg)	Recovery/%	RSD/%
50	51.30	103	5.0
500	486.40	97.3	4.2
5000	4932.50	98.7	4.6

## 3 结论

本文利用本实验室构建的水凝胶SERS芯片以及便携式拉曼光谱仪建立了一种快速筛查和检测DON的SERS方法。该方法具有响应范围宽、灵敏度高、重复性好、响应迅速、操作简单等优点,在粮食中生物毒素的快速筛查与检测方面具有良好的应用潜力。
